# Halogen-Mediated Partial Oxidation of Polyvinyl Alcohol for Tissue Engineering Purposes

**DOI:** 10.3390/ijms21030801

**Published:** 2020-01-25

**Authors:** Silvia Barbon, Elena Stocco, Daniele Dalzoppo, Silvia Todros, Antonio Canale, Rafael Boscolo-Berto, Piero Pavan, Veronica Macchi, Claudio Grandi, Raffaele De Caro, Andrea Porzionato

**Affiliations:** 1 Department of Neurosciences, Section of Human Anatomy, University of Padova, Via A. Gabelli 65, 35121 Padova, Italy; silvia.barbon@unipd.it (S.B.); elena.stocco@gmail.com (E.S.); rafael.boscoloberto@unipd.it (R.B.-B.); veronica.macchi@unipd.it (V.M.); andrea.porzionato@unipd.it (A.P.); 2 L.i.f.e.L.a.b. Program, Consorzio per la Ricerca Sanitaria (CORIS), Veneto Region, Via N. Giustiniani 2, 35128 Padova, Italy; 3 Department of Pharmaceutical and Pharmacological Sciences, University of Padova, Via F. Marzolo 5, 35128 Padova, Italy; dalzoppodaniele@gmail.com (D.D.); claudio.grandi@unipd.it (C.G.); 4 Department of Industrial Engineering, Centre for Mechanics of Biological Materials, University of Padova, Via Venezia 1, 35131 Padova, Italy; silvia.todros@unipd.it (S.T.); piero.pavan@unipd.it (P.P.); 5 Department of Statistical Sciences, University of Padova, Via C. Battisti 241, 35121 Padova, Italy; antonio.canale@unipd.it; 6 Fondazione Istituto di Ricerca Pediatrica Città della Speranza, 35121 Padova, Italy; 7 Foundation for Biology and Regenerative Medicine, Tissue Engineering and Signaling (T.E.S.) Onlus, 35030 Padova, Italy

**Keywords:** polyvinyl alcohol, chemical oxidation, halogens, scaffold, tissue engineering

## Abstract

Partial oxidation of polyvinyl alcohol (PVA) with potassium permanganate turned out to be an efficient method to fabricate smart scaffolds for tissue engineering, endowed with biodegradation and protein delivery capacity. This work considered for the first time the use of halogens (bromine, chlorine and iodine) as less aggressive agents than potassium permanganate to perform controlled PVA oxidation, in order to prevent degradation of polymer molecular size upon chemical modification. Oxidized PVA solutions were chemically characterized (i.e., dinitrophenylhydrazine assay, viscosity measurements, molecular size distribution) before preparing physically cross-linked hydrogels. Scaffolds were assessed for their mechanical properties and cell/tissue biocompatibiliy through cytotoxic extract test on IMR-90 fibroblasts and subcutaneous implantation into BALB/c mice. According to chemical investigations, bromine and iodine allowed for minor alteration of polymer molecular weight. Uniaxial tensile tests demonstrated that oxidized scaffolds had decreased mechanical resistance to deformation, suggesting tunable hydrogel stiffness. Finally, oxidized hydrogels exhibited high biocompatibility both in vitro and in vivo, resulting neither to be cytotoxic nor to elicit severe immunitary host reaction in comparison with atoxic PVA. In conclusion, PVA hydrogels oxidized by halogens were successfully fabricated in the effort of adapting polymer characteristics to specific tissue engineering applications.

## 1. Introduction

The functional regeneration of damaged or diseased tissues and organs remains an intriguing challenge for modern medicine [1]. Advances in the research field on regenerative biomaterials have inspired successful strategies for the development of smart, affordable and safe scaffolds which promote the restore of tissue form and functionality [2]. However, tissue engineering investigation is constantly in search of an ideal customizable scaffold to better fit the specific features of the tissue to be repaired [3,4,5,6,7]. In this context, synthetic biodegradable polymers, such as polylactic acid, polyglycolic acid, polycaprolactone and polylactide-co-glycolide, offer unique physical, chemical, biological, and biomechanical properties to provide efficient regenerative therapy [8]. Among other polymers, polyvinyl alcohol (PVA) has been increasingly appreciated in medical applications as a biocompatible, atoxic and non-immunogenic biomaterial which seems to present ideal characteristics for the preparation of implantable devices (i.e., tunable mechanical and drug delivery properties) [9,10,11,12]. Nevertheless, in the form of a cross-linked hydrogel, it presents poor biodegradation capacity [13]. To overcome this limit, partial oxidation of PVA has been widely investigated as a post-modification of the polymer finalized at enhancing its biodegradation profile, mainly for ecological disposal purposes [14]. Currently, different oxidation processes of PVA are described in literatures. In 2001, Won and Colleagues [15] reported PVA degradation by polymer incubation in water at 200 °C, in the presence of oxygen. In the work described by Zimin et al. [16], mixtures of ozone/oxygen and oxygen/oxygenated water are investigated to study PVA radical oxidation kinetics. An oxidation strategy based on the treatment with persulphate in the presence of 365 nm-UV light was reported by Chia-Chang and Collaborators [17]. The study showed that the oxidative modification is influenced by the pH value, being more efficient in the presence of acid pH rather than alkaline pH. The oxidation kinetics of PVA with potassium permanganate was investigated by Maqsood et al. [18].

Our research group considered for the first time the chemical oxidation of PVA to render it a biodegradable scaffold for tissue engineering applications [19]. Through partial oxidation with potassium permanganate (KMnO_4_) in perchloric acid (HClO_4_), we manufactured a modified PVA hydrogel that is more prone to in vivo degradation. In addition, the oxidation of the hydroxyl groups to carbonyl groups allowed to introduce into the polymer chain more reactive chemical functionalities, which can be used for binding drugs or bioactive growth factors [19,20,21]. Intriguingly, when scaffolds made of PVA oxidized with KMnO_4_ were prepared in the form of hollow nerve conduits or nerve wraps and implanted into a rat model of sciatic nerve resection, they demonstrated to efficiently promote peripheral nerve regeneration, as well as confirming biocompatibility and low immunogenicity of the material [22,23].

Despite the obtainment of a biodegradable and functionalizable scaffold, oxidation of PVA through KMnO_4_ seemed to cause a loss of polymer viscosity, which is strictly correlated to material degradation for the decrease of its mean molecular weight [24]. We hypothesized that this could be ascribed to the poor stereoregularity of the commercial polymer, which causes the addition of a certain percentage of head-to-head monomers (with resulting 1,2-diols) during polymerization. In this case, the treatment with permanganate in acid environment could result not only in material oxidation, but also in the non-selective cleavage of C-C bonds of 1,2-diols in the PVA chain, leading to the decrease of the mean molecular weight of the polymer. Starting from these considerations, the present work investigated for the first time the possibility to perform controlled PVA oxidation by using halogens [i.e., bromine (Br_2_), chlorine (Cl_2_) and iodine (I_2_)] as less aggressive chemical agents, which could assure for the minor degradation of polymer molecular size. Thus, it will be possible to standardize different polymer oxidation conditions to produce customizable PVA scaffolds for specific tissue engineering applications.

## 2. Results

### 2.1. Quantification of 1,2-Diol Content in Neat PVA

To confirm the hypothesis that commercial PVA may contain head-to-head units which can be unselectively cleaved during oxidation process, we quantified the 1,2-diols in the PVA backbone by reaction with sodium periodate (NaIO_4_). This specific reagent selectively cleaves the C-C bond of 1,2-diols, generating two aldehyde functionalities and reducing to iodate (IO_3_^−^) ion. If this effectively occurs, a decrease of polymer molecular weight is expected.

**Figure 1:**
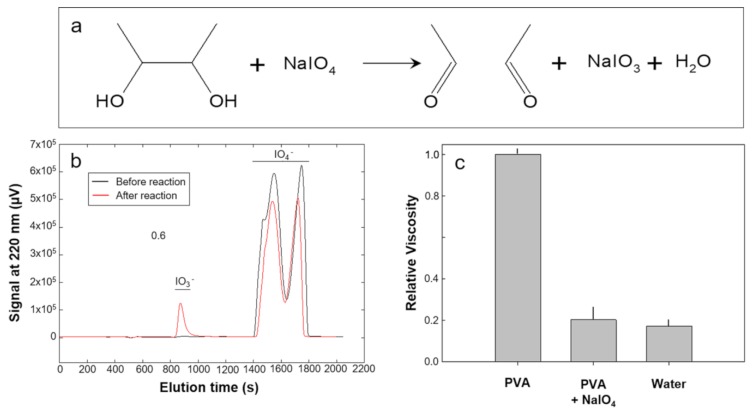
1,2-diol content and relative viscosity of PVA. (**a**) Reaction between 1,2-diols and NaIO_4_, leading to the cleavage of the single bond connecting the two carbon atoms carrying the hydroxyl groups. (**b**) Separation by ion-pairing HPLC of periodate (IO_4_^−^) and iodate (IO_3_^−^) ions before and after reaction with polyvinyl alcohol (PVA). Interestingly, the elution profile of IO_4_^−^ ion is complex, probably because the amount of modifier cetyltrimethylammonium chloride is not enough to transform the IO_4_^−^ species in a single type of ionic couple. (**c**) Relative viscosity of a solution containing 2% (w/w) PVA in water after reaction with NaIO_4_.

After reaction, the single bond connecting the two carbon atoms carrying the hydroxyl groups remains cleaved, as reported in the scheme on Figure 1a. Therefore, after the reaction with periodate, a reduction of the mean molecular weight of the polymer is expected, that can be observed through viscosity measurements. Accurate quantitative measurement by High Performance Liquid Chromatography (HPLC) of the amount of produced sodium iodate (NaIO_3_) (Figure 1b) allowed quantitation of 1,2-diols group content in the commercial PVA used for this work. We estimated a 1,2 diols content of about 1.8%, that justifies the dramatic decrease of viscosity of the PVA solution after reaction with NaIO_4_, as shown in Figure 1c. The initial mean molecular weight of neat PVA, between 124,000 and 184,000 Da, would be reduced, on the basis of a 1.8% 1,2-diols content, to a range between 2000 and 2900 Da.

### 2.2. Partial Oxidation of PVA

Partial oxidation of PVA was carried out through four different oxidizing agents: KMnO_4_ in dilute HClO_4_; bromine (Br_2_), chlorine (Cl_2_) and iodine (I_2_) in sodium bicarbonate (NaHCO_3_) buffer solution. The polymer powder dissolved in water was treated with the stoichiometric quantity of oxidizing agent sufficient to oxidize the 1% or 2% of secondary alcoholic groups to carbonyl groups. The obtained oxidized materials were: (a) 1% and 2% oxidized PVA with KMnO_4_ (OxPVA_KMnO_4__1 and OxPVA_KMnO_4__2, respectively); (b) 1% and 2% oxidized PVA with Br_2_ (OxPVA_Br_2__1 and OxPVA_Br_2__2, respectively); (c) 1% and 2% oxidized PVA with Cl_2_ (OxPVA_Cl_2__1 and OxPVA_Cl_2__2, respectively); (d) 1% and 2% oxidized PVA with I_2_ (OxPVA_I_2__1 and OxPVA_I_2__2, respectively).

The chemical schemes of different reactions for PVA oxidation are described in Table 1.

### 2.3. Analysis of Carbon-13 (^13^C) by Nuclear Magnetic Resonance (NMR) Spectroscopy

**Figure 2:**
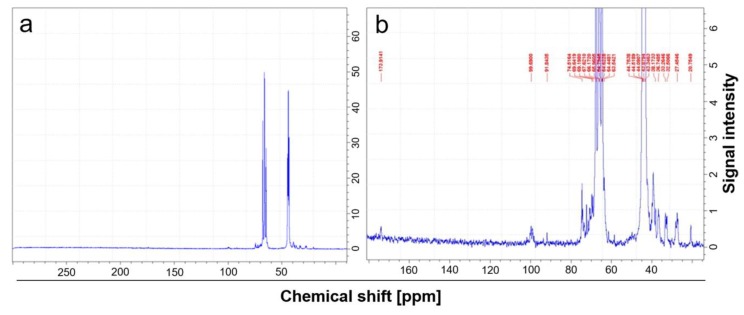
NMR analysis of ^13^C in oxidized PVA. ^13^C-NMR spectrum of oxidized PVA sample in deuterated water. (**a**) Less expanded spectrum, where the intense peaks correspond to carbons of the methylene and methine groups. (**b**) Highly amplified spectrum, where the weak signal at 179 ppm can be attributed to carbons of the carbonyl groups, while the signals at about 99 ppm could be due to hemiacetal or acetal carbons. The spectra are representative of all the types of oxidized PVA.

In order to verify the effective introduction of carbonyl groups in PVA backbone after partial oxidation, all oxidized samples were examined by ^13^C-NMR. To have a detectable signal by carbonyl groups, it was necessary to prepare a concentrate solution (160 mg/mL) in deuterated water and to make more than 40,000 transient acquisitions before carrying out the Fourier transform of the signal. Figure 2b shows an expansion of the ^13^C spectrum, indicating the sharp peak that can be attributed to carbonyl (ketonic groups) and the broad bands probably due to hemiacetals and acetals. The signals are very small compared to the huge amount of ^13^C deriving from the methylene and methine groups of the PVA main chain. This type of NMR spectra does not allow a quantitative evaluation of carbonyl peak.

### 2.4. Partial Oxidation Assessment and Moisture Content

NMR spectroscopy was not sensitive enough to quantify the low percentages (1% and 2%) of carbonyl groups introduced into PVA by chemical oxidation. Thus, the percentage of carbonyl groups in the polymer backbone was determined by 2,4-dinitrophenylhydrazine (DNPH) assay and directly corresponds to the oxidation level of the modified polymer (Table 2). After lyophilization, the four types of oxidized PVA under investigation were obtained in a lyophilized form, appearing as white spongeous material with typical sericeous reflexes. Starting from each lyophilized PVA, the moisture content was determined weighing a small quantity of polymer before and after heating in a current of dry nitrogen for about one hour. Table 2 summarizes the experimental results.

### 2.5. Relative Viscosity of Oxidized PVA

**Figure 3:**
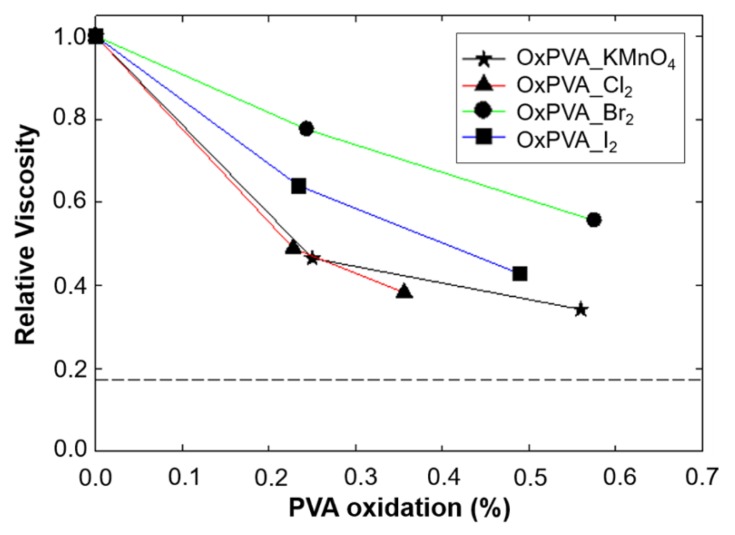
Viscosity measurements after PVA oxidation. Relative viscosity of oxidized PVA solutions was measured after dissolving 2% (w/w) of polymer into water at 20 °C and using a solution of 2% neat PVA as a reference. The dotted line represents relative viscosity of water.

Solution viscosity is correlated with shape and dimension of the molecules dissolved in a solvent. In this study, oxidized PVA was dissolved into water at a concentration of 2% (w/w) and relative viscosities were measured using a solution of 2% neat PVA as a reference. In the hypothesis that shape of molecules of PVA and oxidized PVA is similar, a decrease of the viscosity after oxidation process would indicate a diminution of molecular weight of the molecules. Figure 3 reports the relative viscosities of oxidized PVA solutions.

### 2.6. Elution Profiles of Neat and Oxidized PVA

Molecular dimension and distribution of PVA partially oxidized with bromine and iodine was investigated by high performance gel permeation chromatography (HPGPC) using a Superose™ 6 column. Regarding OxPVA_Cl_2_, viscosity measurements clearly demonstrated a molecular degradation of the polymer comparable to that of OxPVA_KMnO_4_, so it was not considered for further gel filtration analysis.

Oxidized PVA solutions are not colored and their light absorption is weak in the far ultraviolet. Thus, in order to allow an easier detection in the near ultraviolet region, the partially oxidized PVA solutions were covalently labeled with high absorbing molecules like para-aminobenzoic acid (PABA) or DNPH, taking advantage of the presence of carbonyl chemical groups. 

**Figure 4:**
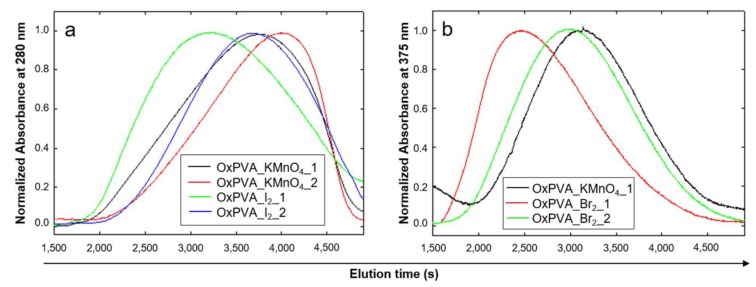
HPGPC of oxidized PVA. After labeling with PABA (**a**) or DNPH (**b**), the elution profiles of oxidized PVA were obtained by chromatography on a Superose 6TM column. The profiles of the bands were normalized to facilitate the comparison.

Labeling with PABA was realized at alkaline pH in sodium bicarbonate and the intermediate Schiff-base was reduced selectively with sodium cyanoborohydride. Since the extremely intense absorption of PABA can overlap and blind the band due to decorated PVA, after labeling reaction PABA excess was removed with a Sepharose G10 column before performing HPGPC for oxidized PVA evaluation. Figure 4 reports the normalized elution profile of oxidized PVA after labeling with PABA, which was successful only for the PVA partially oxidized with permanganate and iodine. On the contrary, the PVA oxidized with bromine coagulated in the conditions adopted for PABA labeling.

Lower elution times in GPC corresponds to higher molecular weights; as shown by the elution profiles reported in Figure 4a, OxPVA_I_2__1 and OxPVA_I_2__2 maintain a mean molecular weight which appears to be superior to permanganate-partially oxidized PVA. 

OxPVA_Br_2__1 and OxPVA_Br_2__2 were labeled with DNPH, the same reagent and the same reaction conditions adopted for the evaluation of carbonyl content. Additionally in this case, the excess of DNPH was removed by chromatography on Sephadex^®^ G-25 column prior to HPGPC. The elution profiles of OxPVA_Br_2__1 and OxPVA_Br_2__2 obtained by chromatography on Superose™ 6 are reported in Figure 4b, in comparison with the profile produced by OxPVA_KMnO_4__1 previously labeled with DNPH. As for iodine-based oxidation, also the use of elemental bromine seemed to produce an oxidized polymer with higher molecular weight in comparison to PVA partially oxidized with KMnO_4_.

### 2.7. Mechanical Properties

**Figure 5:**
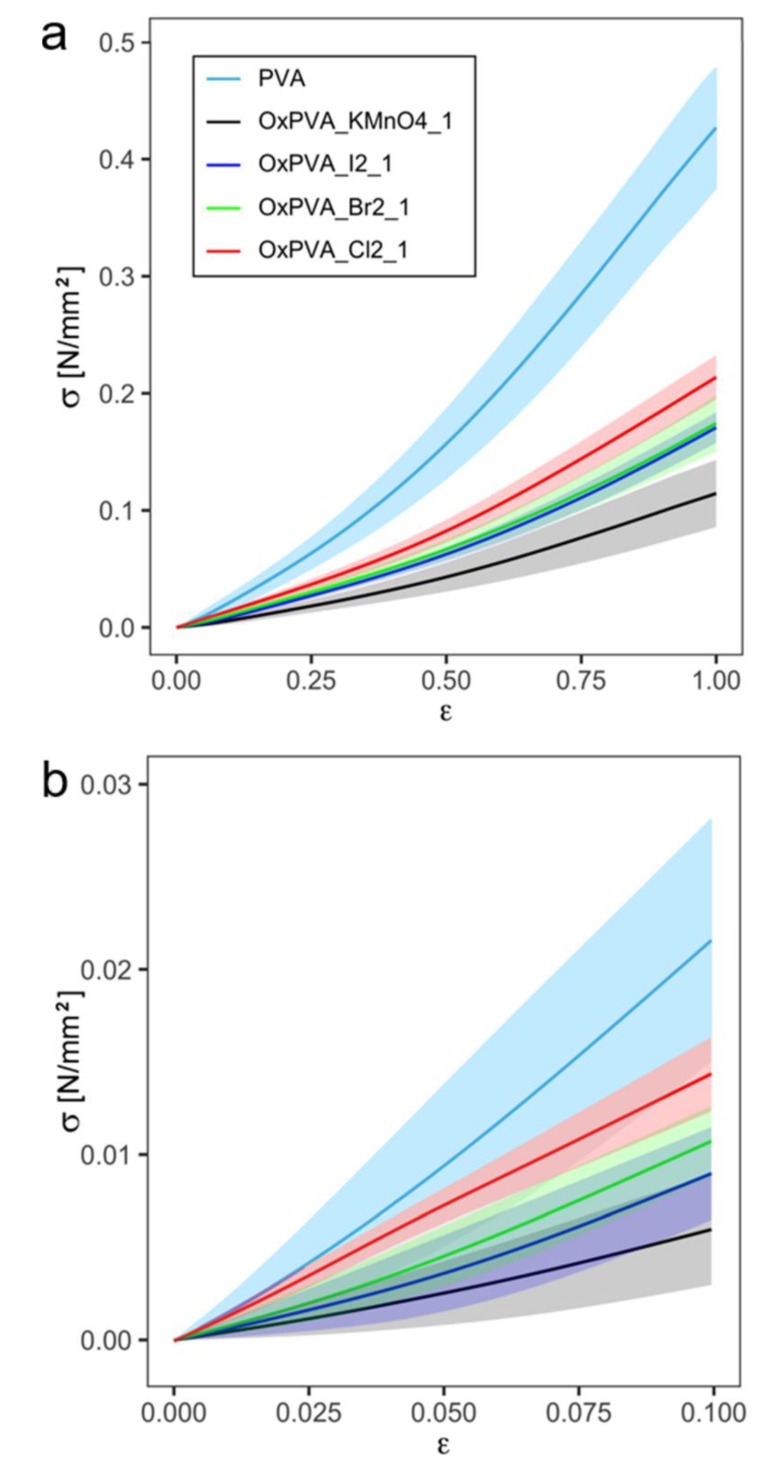
Mechanical behavior of PVA. Results of uniaxial tensile tests of PVA and oxidized PVA, reported in terms of nominal stress σ [N/mm^2^] vs. nominal strain ε, on the overall test range (up to 100% strain) (**a**) and in a smaller strains range (between 0 and 10% strain) (**b**).

The tensile behavior of neat and oxidized PVA is described by Figure 5, where functional means curves (continuous lines) are reported for each material, along with variability bands (shaded areas) obtained adding and subtracting the functional standard deviations. All samples showed a non-linear behavior with stiffening at increasing strain and without break in the overall strain range selected for tensile testing.

The values of the secant elastic modulus E_s_ at 10% and 20% strain are reported in Table 3 for each material. A difference between neat and oxidized PVA is shown in Figure 5, particularly for higher strain values. The discretized *p*-value function suggests strong evidence against the null hypothesis (the groups are equal), since *p*-value < 0.01 all over the strain range, except obviously from a neighborhood of the left bound of the domain where all the samples are undergoing null stress.

The results of Bonferroni post-hoc test, reported in Table 3, showed a statistical difference between secant elastic modulus E_s_ of neat PVA and all oxidized PVA hydrogels at both 10% and 20% strain. Moreover, E_s_ of OxPVA_Cl_2__1 is significantly different from E_s_ of OxPVA_KMnO_4__1 and OxPVA_I_2__1 at both strain values. In addition, a significant difference was found between E_s_ of OxPVA_KMnO_4__1 and OxPVA_Br_2__1 at 20% strain.

### 2.8. Polymer Biocompatibility

**Figure 6:**
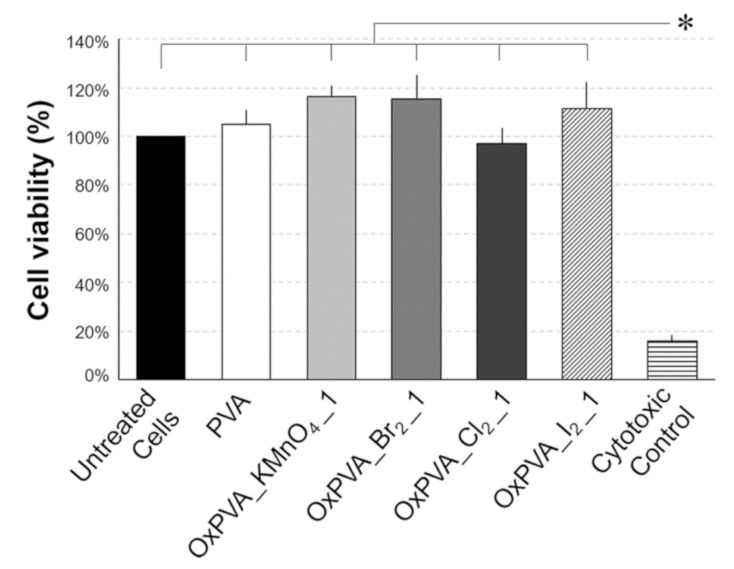
Cytotoxicity extract test. Relative viability of IMR-90 cells treated with neat and oxidized PVA-conditioned media in comparison with the negative (untreated cells) and the cytotoxic (50% DMSO) controls (*: *p* ≤ 0.01).

Testing the effects of synthetic polymers on the viability of cells grown in culture is widely used as a predictor of potential toxic effects in the perspective of in vivo implant studies. The cytotoxicity extract test demonstrated that neat and oxidized PVA-conditioned media did not affect IMR-90 cell viability in comparison with basal culture conditions (Figure 6). Altogether, no significative difference between different oxidized PVA hydrogels was observed with regard to cytotoxicity, with calculated cell viabilities of 105 ± 6% (PVA), 116 ± 5% (OxPVA_KMnO_4__1), 115 ± 10% (OxPVA_Br_2__1), 97 ± 7% (OxPVA_Cl_2__1), 112 ± 11% (OxPVA_I_2__1), setting to 100% the viability of untreated control cells. Moreover, both untreated and treated samples showed significatively preserved cell viability compared to the cytotoxic control, where the percentage of living cells was reduced to 16 ± 2%, referring to the untreated control (100%).

**Figure 7:**
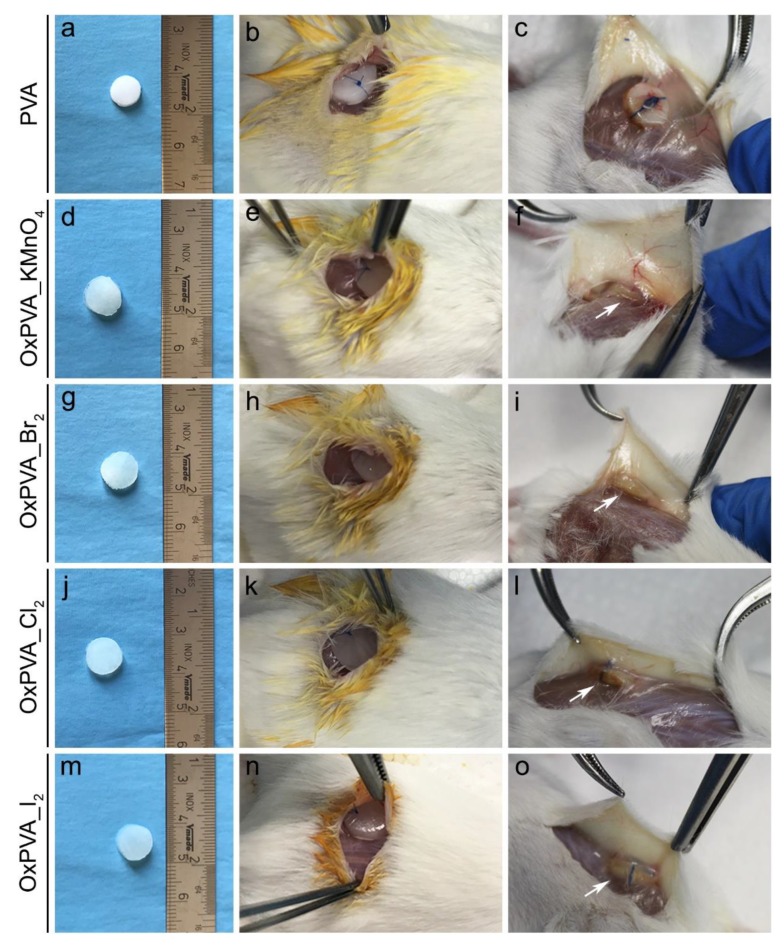
Macroscopic evaluation of implanted PVA scaffolds. Gross appearance of neat and oxidized PVA hydrogel disks before implantation into BALB/c mice (**a**,**d**,**g**,**j**,**m**), after insertion into a dorsal subcutaneous pouch of the animals (**b**,**e**,**h**,**k**,**n**) and at the time of explantation, 4 weeks after surgery (**c**,**f**,**i**,**l**,**o**).

Finally, the biocompatibility of PVA-derived scaffolds was also assessed in vivo, by implantation in the subcutaneous dorsal region of BALB/c mice for 4 weeks (Figure 7). Macroscopic evaluation of samples at the time of explanation showed that they were still well recognizable at the implant site, being surrounded by a thin connective tissue capsule. Scaffolds did not show any alteration of dimensions and gross morphology, with no evidence of polymer degradation during the four week-implantation period. Remarkably, there were no signs of infection or rejection of the graft (Figure 7).

**Figure 8:**
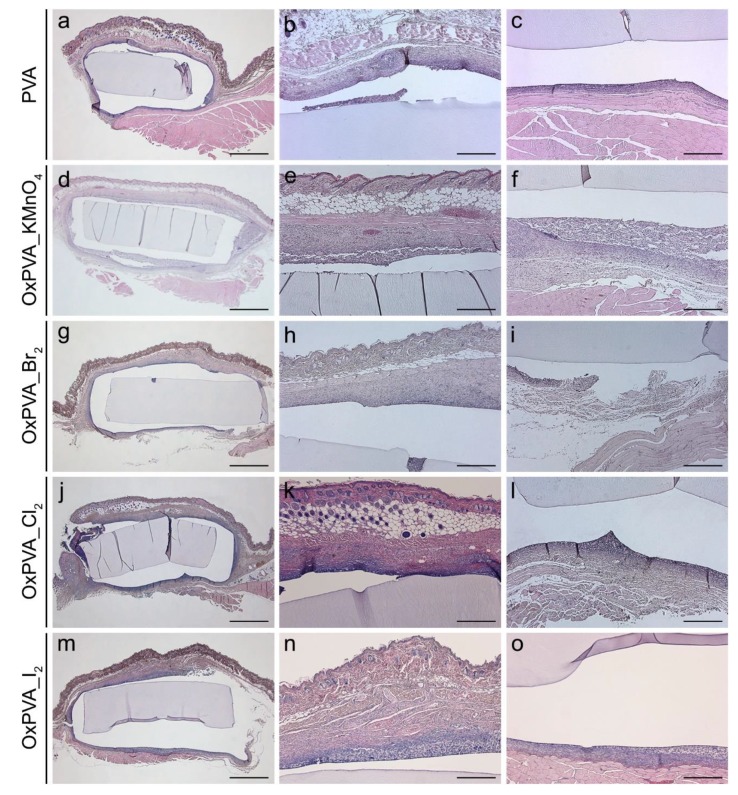
Histological evaluation of explanted PVA scaffolds. Hematoxylin and eosin staining of neat and oxidized PVA scaffolds after 4 weeks of subcutaneous implantation into the dorsal region of BALB/c mice. All hydrogel disks could be well identified among the surrounding tissues (**a**,**d**,**g**,**j**,**m**) and no severe inflammatory infiltration was observed at the subcutaneous (**b**,**e**,**h**,**k**,**n**) and muscular (**c**,**f**,**i**,**l**,**o**) sides. Scale bars: (**a**,**d**,**g**,**j**,**m**) 100 µm; (**b**,**c**,**e**,**f**,**h**,**i**,**k**,**l**,**n**,**o**) 400 µm.

In histological sections stained with hematoxylin and eosin, PVA-derived scaffolds could be well identified among the surrounding host tissues, with no degradation changes affecting the microscopic morphology. Moreover, no severe inflammatory infiltrate was detected neither in the cutaneous nor in the muscular side (Figure 8).

**Figure 9:**
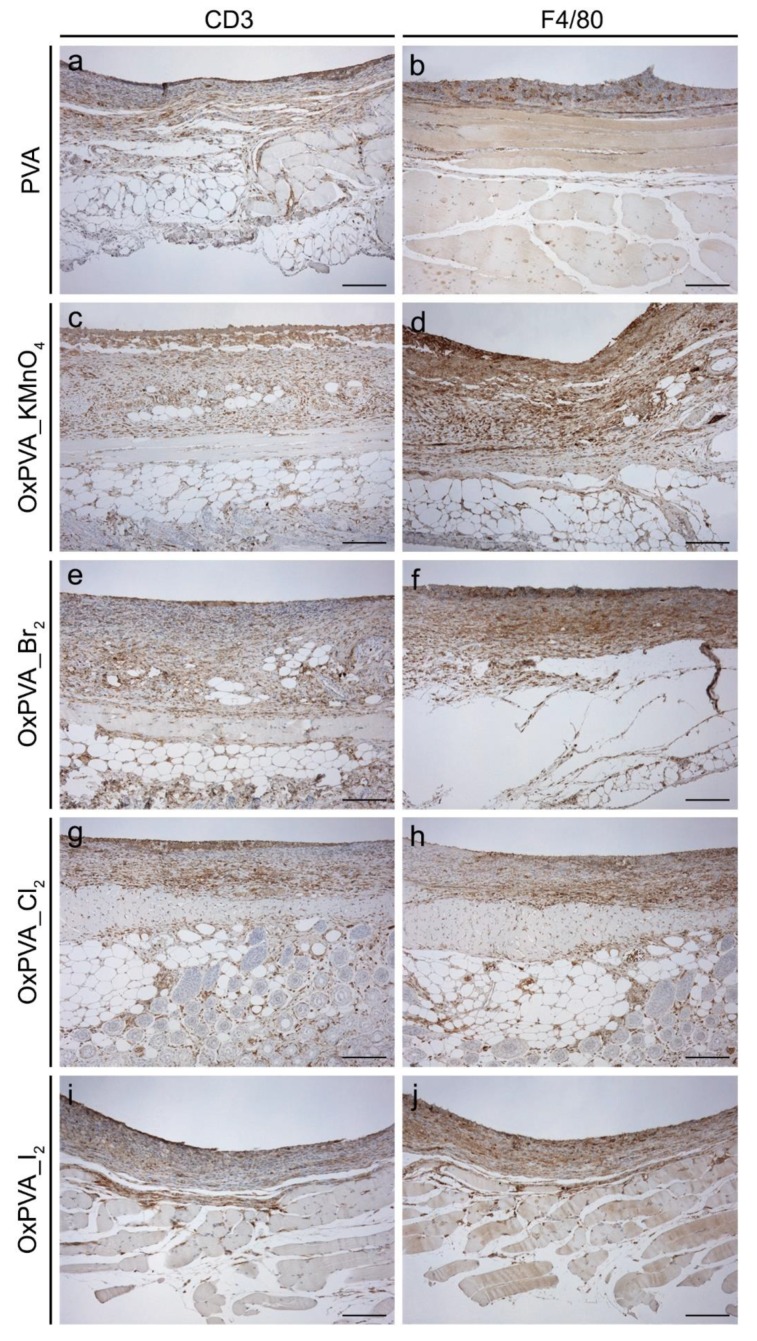
Lympho-monocytic cell infiltration. Immunolocalization of CD3+ lymphocytes (**a**,**c**,**e**,**g**,**i**) and F4/80+ macrophages (**b**,**d**,**f**,**h**,**j**) on explanted samples after 4 weeks of subcutaneous implantation into BALB/c mice. Only a slight infiltration of inflammatory cells was observed into tissues surrounding PVA-derived scaffolds, confirming the biocompatibility of the polymer after oxidation treatment. Scale bar: 150 µm.

Immunolocalization of the lympho-monocytic cell fraction confirmed the low immunogenicity of both neat and oxidized PVA hydrogels. Figure 9 showed that no serious inflammatory reactions were detected, revealing only a slight infiltration of the connective tissue surrounding the implanted material.

## 3. Discussion

In the effort of improving the biological features of PVA hydrogel, our previous research focused on two main aspects. Firstly, we successfully fabricated composite scaffolds by combining the polymer with bioactive extracellular matrices (ECMs). Decellularized, homogenized and lyophilized sheets from articular cartilage, Wharton’s Jelly and small intestine were cross-linked to PVA, obtaining bio-hybrid supports that sustained cell adhesion and proliferation [25,26,27]. In parallel, we proposed for the first time a standardized method to perform chemical oxidation of PVA by KMnO_4_ in acid environment, in order to obtain scaffolds with improved swelling kinetics and protein adsorption/release ability with respect to neat PVA, as well as with tunable biodegradation properties which increase along with the degree of oxidation [19,22,23].

Although OxPVA_KMnO_4_ proved to possess encouraging properties to be used as a bioabsorbable scaffold for TE applications, it resulted to suffer from lower viscosity than non-modified PVA, reveling some alteration of polymer molecular weight due to the oxidation process. To explain this, we considered that the commercial PVA comes from the hydrolysis of polyvinyl acetate (PVAc), which is obtained from the radical polymerization of the monomer vinyl acetate (Vac). The control of radical polymerization stereoselectivity is not easily performed, resulting into the random addition of head-to-head monomers instead of the prevalent head-to-tail insertions in the polymer chain. This implies that, after hydrolysis with removal of the acetate group, the PVA contains some 1,2-diol groups which could be easily cleaved during the oxidation process with KMnO_4_, causing a decrease of polymer molecular weight. Our hypothesis was confirmed by demonstrating the production of IO_3_^−^ ion after reaction of PVA solution with NaIO_4_, which caused the selective cleavage of the C-C single bond carrying the hydroxyl groups. This allowed us to quantify the 1,2-diol content in the polymer, in order to estimate a decrease of PVA mean molecular weight from 154,000 Da to about 2400 Da.

To support this result, polymer viscosity (i.e., molecular weight) was found to substantially decrease after reaction of PVA solution with NaIO_4_. Since the literature reports that the oxidation with KMnO_4_ in acid environment could potentially cause the cleavage of the C-C bond of 1,2-diols [28] (which we proved to be present in the PVA chain), we deduced that the decrease in viscosity of PVA solution after treatment with this oxidizing agent may arise from main-chain cleavage at head-to-head units with consequent alteration of polymer molecular size.

Starting from these considerations, this work aims at identifying novel oxidizing agents which can assure for the obtainment of partially oxidized PVA with the minor variation in molecular weight distribution compared to the starting polymer. Halogens as Br_2_, Cl_2_ and I_2_ possess standard oxidation-reduction potential which is sufficient to oxidize secondary alcohol groups of PVA polymer chain to ketone groups. To the best of our knowledge, PVA oxidation mediated by halogens has never been considered, except for a work by Ye and Collaborators [29], which described a method for UV/Cl_2_ oxidation of PVA to promote its degradation for ecological disposal purposes. Thus, here we standardized a method to produce and investigate novel PVA hydrogels partially oxidized with Br_2_, Cl_2_ and I_2_, in the perspective of using them as synthetic scaffolds for tissue regeneration. In parallel, OxPVA_KMnO4 was considered as a reference. Given the paucity of experimental data about halogen-mediated PVA oxidation and considering that Br_2_, Cl_2_ and I_2_ possess different redox potentials, we decided to test all the three oxidizing agents for the effect they can produce on polymer modification.

After standardization of the oxidation method, the determination of carbonyl groups in all the types of oxidized PVA was first performed by infrared (IR) spectroscopy. Unfortunately, this technique turned out to be not sensitive enough to detect the small amount (1% and 2%) of carbonyl groups introduced into the polymer after oxidation. In fact, IR-spectra of neat and oxidized PVA were found nearly to overlap, not allowing an accurate and reproducible quantification of carbonyl content. Given that, oxidized PVA was subsequently analyzed by NMR spectroscopy to detect the ^13^C of the carbonyl groups. Additionally in this case, the carbonyl signal was very small. However, the presence of other weak signals suggested that most of the carbonyls formed by oxidation were probably converted into hemiacetal and acetal groups.

Since both IR and NMR spectrometry gave no satisfactory results, we decided to perform DNPH assay as a more accurate and reliable method to estimate polymer oxidation. DNPH assay effectively allowed to quantify the carbonyl content of the partially oxidized PVA, confirming that chemical modification with all oxidants efficiently occurred.

The effect of halogen-mediated oxidation on PVA polymerization or depolymerization was then assessed by determining polymer solution relative viscosity, which results to be a good practical measure of polymer molecular weight [24]. The analysis revealed that Br_2_ and I_2_ caused the minor loss of PVA viscosity, turning out to be milder oxidants than Cl_2_ and KMnO_4_ and probably less prone to carbon-carbon cleavage of 1,2-diols, assuring for minor alteration of PVA molecular weight. Comparing the oxidation-reduction potentials of the three halogens, the mildest oxidant results to be I_2_. In practice, however, the oxidation with I_2_ needs to be carried out at a higher temperature (i.e., 70 °C) to avoid the formation of I_2_/PVA complexes which would lead to an extended agglutination of the polymer. Since I_2_ was not found to cause the minor alteration of PVA viscosity, we can infer that the elevated temperature derived the reaction over activation barriers necessary for 1,2-diols cleavage, facilitating their oxidative fragmentation [30]. On the contrary, both Br_2_ and Cl_2_ do not form agglutinating complexes with PVA at low temperatures and showed different effects on polymer solution viscosity upon oxidation. In particular, Cl_2_, being the strongest oxidizing agent, resulted to cause a loss of PVA viscosity which was comparable to that observed for KMnO_4_-mediated oxidation. 

Since viscosity measurements indicated a certain viscosity alteration of all oxidized polymer solutions, HPGPC was performed to compare molecular weight distribution among different samples by using a different procedure. Unfortunately, it was not possible to find polymers with hydrodynamic behavior similar to PVA and with known molecular weight distribution to serve as standards for gel filtration column calibration. This prevented us from quantifying the range of molecular weights of the analyzed PVA solutions. After PVA labeling with DNPH and PABA in order to make them visible with the spectrophotometric detector, gel chromatography analysis confirmed viscosity measurements results, highlighting that Br_2_ is the most selective oxidant and allowed to obtain partially oxidized PVA which maintains a molecular weight distribution similar to that of neat PVA.

Aiming at using novel oxidized PVA hydrogels as implantable scaffolds for tissue regeneration, we investigated how the oxidation process affected polymer mechanical behavior. After uniaxial tensile test, a non-linear stiffening behavior of neat and oxidized PVA was measured in the overall strain range selected in this investigation. This strain range (up to 100%) is extremely higher than physiological range which any biomaterial could experience in vivo. However, experimental results pointed out that the materials did not show any failure or permanent strain at the maximum strain applied, recovering the initial length in the unloading phase. This characteristic is important when it is necessary to ensure a safety factor for the strength of a material. In the perspective of future TE applications, the first part of stress-strain response is worth investigating. As shown by the values of the secant elastic modulus, neat PVA resulted to be stiffer than oxidized PVA. In particular, the oxidation with KMnO_4_ induced the strongest decrease in stiffness, while the oxidation with Cl_2_ less intensely affected PVA stiffness. The oxidation with I_2_ and Br_2_ provided very similar effects in terms of mechanical properties modification, being OxPVA_Br_2__1 slightly stiffer than OxPVA_I_2__1. However, due to the variance of experimental results, a significant difference among oxidized PVA scaffolds was found in case of OxPVA_Cl_2__1 vs. OxPVA_KMnO_4__1 and OxPVA_I_2__1 at 10% strain, and OxPVA_KMnO_4__1 vs. OxPVA_Br_2__1 and OxPVA_Cl_2__1, as well as OxPVA_Cl_2__1 vs. OxPVA_I_2__1 at 20% strain.

The mechanical investigation developed in this work was limited to the uni-axial tensile behavior of the materials considered. The loading conditions in vivo can be more general and suggest extending the mechanical characterization to other stress modes, for example considering bi-axial tensile tests or compressive tests [31,32]. Furthermore, it would be interesting to evaluate the time-dependent behavior of native and oxidized PVA due to interaction between solid matrix and permeating liquid phases [33,34]. 

Finally, cellular and tissue compatibility of partially oxidized PVA was studied by in vitro and in vivo models to assess polymer interactions with both human fetal fibroblast cells and mice subcutaneous tissue. In fact, the chemical oxidative treatment might affect PVA atoxicity in terms of possible residual reagents which would be biologically harmful to tissues and cells [35].

First of all, our protocol for neat/oxidized PVA hydrogel preparation starting from the corresponding solutions is based on physical cross-linking by freezing/thawing. This allows to avoid the use of any toxic chemicals, such as cross-linkers and initiators (i.e., glutaraldehyde [13]), which would augment the risk of alter polymer cytocompatibility. The extract cytotoxicity test highlighted that IMR-90 fibroblasts cultured with oxidized PVA-conditioned media preserved cell viability as much as untreated cultures and cells grown into medium conditioned with known atoxic PVA.

In addition to the in vitro cytotoxicity studies, the biocompatibility of oxidized scaffolds in vivo was also tested. A subcutaneous implantation model using BALB/c mice was investigated to obtain preliminary results of in vivo biocompatibility in the perspective of scaffold orthotopic implant studies. Animal experiment corroborated in vitro cell culture assay, demonstrating that all types of oxidized PVA hydrogels maintained high biocompatibility and low immunogenicity in comparison with neat PVA, which was reported to be atoxic and biocompatible. These results confirmed that the prolonged dialysis of the polymer solutions after oxidation assured for the elimination of any potentially toxic residual products derived from the chemical modification process. Further in vivo experiments of subcutaneous implantation for longer end points (i.e., up to 12 weeks) will be necessary to define the biodegradation profile of oxidized PVA-derived scaffolds.

Overall, this study on the halogen-mediated oxidation of PVA pointed out that Br_2_ and I_2_ can effectively modify the polymer assuring a better preservation of molecular weight distribution in comparison with KMnO_4_, with Br_2_ producing the best outcome. The possibility to control the polymeric structure of oxidized PVA could have an impact on its specific biological features. In this work, we found that, in comparison with KMnO_4_, halogens as Br_2_ can produce oxidized scaffolds with higher mechanical stiffness, suggesting that they can be preferred to restore tissue districts where higher mechanical resistance is required. Furthermore, the preserved molecular weight distribution of PVA oxidized by halogens can ensure for a more homogeneous network structure of the polymer, which may affect the release kinetics of proteins/drugs absorbed by the scaffold. However, these biological differences among oxidized PVA scaffolds need to be evaluated with specific experimental approaches to determine which oxidized hydrogel can be used in specific clinical applications. 

## 4. Materials and Methods

### 4.1. Reagents

PVA [Molecular Weight (MW) 124,000-184,000 Da], phosphate buffered saline (PBS), 70% hydrochloric acid (HCl), 80% phosphoric acid (H_3_PO_4_), perchloric acid (HClO_4_), acetonitrile, elementary Br_2_, elementary Cl_2_, 30% oxygenated water, 32% HCl, sodium periodate (NaIO_4_), sodium bicarbonate (NaHCO_3_), sodium cyanoborohydride, cetyltrimethylammonium chloride, para-aminobenzoic acid (PABA), were supplied by Sigma-Aldrich (S. Louis, MO, USA). KMnO_4_, elementary I_2_, potassium iodide, 2-propanol, ethylene glycol, methanol and dimethyl sulfoxide (DMSO) were purchased from Carlo Erba (Milan, Italy).

### 4.2. Preparation of PVA and Oxidized PVA Solutions

An aqueous solution of 16 wt% neat PVA was prepared as described previously by Stocco and Collaborators [19,25]. In brief, PVA powder was weighted, suspended into milliQ water and then heated for 48 hours (h) at 100 °C under stirring until complete dissolution of the polymer. In parallel, each type of oxidized PVA was obtained by the experimental procedure following described.

#### 4.2.1. PVA Oxidation with Permanganate in Perchloric Acid

The preparation of OxPVA_KMnO_4__1 (1% oxidized PVA with KMnO_4_) was performed according to a published protocol [19]. Briefly, 10 g of PVA were added to 200 g of milliQ water and heated in a boiling water bath for 60 min (min) over a magnetic stirrer. After cooling at 37 °C, the polymer solution was treated with 151 mg of KMnO_4_ in 10 mL of milliQ water and 1.60 g of 70% HClO_4_ (w/w) for 60 min at 37 °C, till complete discoloration. The resulting solution was extensively dialyzed against water using a membrane with 8000 Da cut-off (Sigma-Aldrich). In parallel, the synthesis of OxPVA_KMnO_4__2 (2% oxidized PVA with KMnO_4_) was performed as described above, doubling the amounts of KMnO_4_ and HClO_4_. For long term storage, OxPVA_KMnO_4__1 and OxPVA_KMnO_4__2 solutions were kept at −20 °C overnight to allow complete freezing and then lyophilized using an under-vacuum evaporator (Speed Vac Concentrator Savant, Instruments Inc., Farmingdale, NJ, USA). When experiments had to be carried out, oxidized PVA solutions were recovered by processing lyophilized material as described in Section 4.2 for the neat polymer powder. 

#### 4.2.2. PVA Oxidation with Bromine in Sodium Bicarbonate Buffer Solution

OxPVA_Br_2__1 (1% oxidized PVA with Br_2_) was produced by preparing a solution of PVA powder (5.05 g) into milliQ water (100 g), which was heated in a boiling water bath for 60 min over a magnetic stirrer. The solution was then cooled at 15 °C, before adding 264 mg of NaHCO_3_ and mixing well for complete dissolution. Subsequently, polymer oxidation occurred by treating the solution with 66 µL of Br_2_ in 11 mL of milliQ water for 4 h at 15–18 °C. At the end of the process, a complete clear solution was obtained. Finally, oxidized PVA solution was subjected to dialysis, freezing at −20 °C and lyophilization as described in Section 4.2.1. OxPVA_Br_2__2 (2% oxidized PVA with Br_2_) was obtained following the same procedure, doubling the amounts of Br_2_ and NaHCO_3_.

#### 4.2.3. PVA Oxidation with Chlorine in Sodium Bicarbonate Buffer Solution

The preparation of OxPVA_Cl_2__1 (1% oxidized PVA with Cl_2_) was carried out by weighing 5.05 g of PVA and dissolving the powder into 100 g of milliQ water. The solution was then heated for 60 min in a boiling water bath over a magnetic stirrer. After that, PVA solution was cooled at 15 °C, 287 mg of NaHCO_3_ were added and mixed to the polymer until complete dissolution. The solution was then treated with 7.0 mL of 0.185 M Cl_2_ previously absorbed in 0.30 M phosphate buffer, pH 8.0 and left to react until complete discoloration. Finally, the resulting solution was dialyzed, frozen and lyophilized as previously described. OxPVA_Cl_2__2 (2% oxidized PVA with Cl_2_) was obtained following the same procedure, doubling the amounts of Cl_2_ and NaHCO_3_.

#### 4.2.4. PVA Oxidation with Iodine in Sodium Bicarbonate Buffer Solution

OxPVA_I_2__1 (1% oxidized PVA with I_2_) was prepared by suspending PVA powder (5.05 g) into 100 g of milliQ water and by heating the solution in a boiling water bath for 60 min under agitation. After cooling the solution at 70 °C, 334 mg of NaHCO_3_ were added and mixed until complete dissolution. The solution was then treated with 5 mL of milliQ water containing 315.2 mg of I_2_, dissolved by addition of 574 mg potassium iodide. When the solution was completely clear, it was dialyzed, frozen and lyophilized as previously described. OxPVA_I_2__2 (2% oxidized PVA with I_2_) was obtained following the same procedure, doubling the amounts of I_2_ and NaHCO_3_.

### 4.3. Determination of 1,2-Diol Content in PVA

The amount of 1,2-diols in neat PVA was quantitated through the reaction with an excess of NaIO_4_, which is a fast, selective and quantitative oxidizing agent for 1,2-diols.

For PVA/NaIO_4_ reaction, a glass vial containing 400 mg of PVA and 19.60 g of deionized water were sealed and heated at 100 °C for 30 min until complete dissolution of the polymer. After cooling at room temperature (RT), 10.0 g of the solution were pooled apart and added with 110 mg NaIO_4_ (MW = 213). After 60 min, the viscosity of the treated solution was measured in comparison to the native one. The evaluation of iodate (IO_3_^−^) and periodate (IO_4_^−^) ion content was performed by HPLC, by analyzing an aliquot of 5 µl with a reversed-phase C18 column Bio-Sil ODS-5S (BioRad, Hercules, CA, USA) and the ion-couple reagent cetyltrimethylammonium chloride. The isocratic eluent was constituted by water/acetonitrile 1/1 added with 0.10% H_3_PO_4_ and 1 mM cetyltrimethylammonium chloride. The flow rate was 1.00 mL/min at RT and the spectrophotometric detection was conducted at 220 nm [36]. For calibration of IO_3_^−^ ion, pre-weighed amounts of ethylene glycol were added to five solutions, each containing 10.0 g of water and 110 mg of NaIO_4_. After complete reaction (60 min), 5 µl of each solution were analyzed by HPLC.

### 4.4. Viscosity Measurements

The relative viscosity of aqueous solutions of neat and oxidized PVA was measured using a simple device composed of a reservoir vertically connected to a capillary of fixed length and diameter. The mass of solutions containing 2% (w/w) neat or oxidized PVA in pure water that flowed through the capillary in 240 s (s) was measured at the temperature of 20 °C, starting every time from the same level of the liquid in the reservoir. The reference was always the mass flown of a solution of native PVA of equal concentration. All the solutions were filtered through 1.2 µm pore size cellulose regenerated filters (Merck Millipore, Burlington, MA, USA) to avoid clog or slow down of the liquid flow into the capillary.

### 4.5. High Performance Liquid Chromatography (HPLC)

HPLC analysis was conducted using an LKB system (Uppsala, Sweden) constituted by two pumps model 2248, a high-pressure mixer, a Valco injection valve and a detector multi-wavelength LKB 2141. The management of the chromatographic system and the acquisition of the chromatograms were performed with the software HPLC Manager (Uppsala, Sweden). The elaboration of the chromatograms for quantitative analyses was realized with the software NELSON (PerkinElmer, Waltham, MA, USA). The HPLC system was employed for quantitative analysis of the IO_3_^−^ and IO_4_^−^ ions, for low pressure Sepharose G10, Sepharose G25SF gel permeation chromatography (GPC), as well as for high performance GPC with column Superose™ 6 (GE Healthcare, Chicago, IL, USA).

### 4.6. NMR Analysis of ^13^C in Oxidized PVA

Analyses were performed with a Bruker Avance III nuclear magnetic resonance spectrometer (Bruker BioSpin, Switzerland), operating at 400 MHz of ^1^H frequency and at 100 MHz of ^13^C frequency. Briefly, 80 mg of PVA oxidized with different agents were firstly dried on anhydrous CaCl_2_ under vacuum for 24 h and then inserted into the NMR tube and dissolved with 400 µL of 99% deuterated water (Sigma-Aldrich), heating the tube at 100 °C for 30 min. A total of 40,960 scans were performed to obtain the ^13^C spectrum able to highlight the minimum amount of carbonyls that should be present in the partially oxidized PVA.

### 4.7. Determination of Moisture Content

About 250 mg of each lyophilized oxidized PVA chopped into small pieces were transferred in a test tube partially closed with a perforated rubber stopper. The polymer was heated to 110 °C under a stream of nitrogen (input pressure about 0.2 Bar), flown through a Teflon tube inserted in the stopper. After 70 min, the lyophilized oxidized PVA was removed and weighted.

### 4.8. Evaluation of Oxidized PVA Carbonyl Content through DNPH Assay

Partial oxidation of PVA produces carbonyl groups that can be quantitate by 2,4-dinitrophenylhydrazine (DNPH) (Fluka, Basel, Switzerland) assay. An aliquot of 100 μL was taken from solutions of each polymer and allowed to react with 900 μL of 10 mM DNPH in 2.5 M HCl for 96 h at 20 °C in the dark. The PVA-dinitrophenylhydrazones resulting from the reaction were separated from the excess of DNPH through gel permeation on a Sephadex^®^ G-25 SF column (diameter: 8 mm; length: 12 cm), eluted with PBS/methanol 70/30 (*v*/*v*). The injected volume was 200 μL and flow rate was 0.40 mL/min. The eluate was monitored at 375 nm allowing a manual accurate collection of the peak fraction containing the PVA-dinitrophenylhydrazones. Measuring absorbance at 375 nm and volume of the pooled fraction allowed the quantitative evaluation (molar absorptivity: 22,000 M^−l^ cm^−1^) of carbonyl groups. Some of the collected PVA-dinitrophenylhydrazones were also analyzed by high performance GPC on a Superose™ 6 column as reported subsequently.

### 4.9. Coupling of Para-Aminobenzoic Acid to Oxidized PVA

The coupling reaction of oxidized PVA with PABA was performed in 0.20 M NaHCO_3_, pH 8.2, by mixing 100 μL of 20 mg/mL PABA, 400 μL of oxidized PVA 20 mg/mL solution and 80 μL of 20 mg/mL sodium cyanoborohydride in a 1.5 mL-Eppendorf tube. Reaction was carried on for 48 h at 32 °C and then PABA excess was removed by gel permeation on Sephadex^®^ G-10 column. Briefly, 400 µL of each sample were charged on column and eluted with PBS to a flow rate of 0.40 mL/min. Higher molecular weight PVA covalently bound to PABA eluted first; each peak was collected manually in an Eppendorf tube, monitoring the eluate absorption at 280 nm. Oxidized PVA decorated with PABA were analysed by high performance GPC as reported subsequently.

### 4.10. High Performance gel Permeation Chromatography (HPGPC)

Partially oxidized PVA samples were decorated with PABA or DNPH as previously described, to allow an easy detection through UV absorption. Molecular size dispersion of the polymers was evaluated by HPGPC in water-based eluent using a Superose™ 6 column. In particular, oxidized PVA decorated with PABA were analysed on Superose™ 6 column eluted with 0.25 mL/min PBS. Injection volume was 300 µL and detection wavelength was 280 nm. The column was thermostated to 25 °C. In parallel, collected fractions of oxidized PVA decorated with DNPH were analyzed on Superose™ 6 column eluted with 0.25 mL/min PBS/methanol 70/30 (*v*/*v*). Injection volume was 400 µl and detection wavelength was 375 nm. The column was thermostated to 50 °C.

### 4.11. PVA Hydrogel Preparation

PVA, OxPVA_KMnO_4__1, OxPVA_Br_2__1, OxPVA_Cl_2__1 and OxPVA_I_2__1 hydrogel scaffolds were prepared as already described by Stocco and Collaborators [19,25]. Briefly, a solution of each polymer was poured between two sheets of plate glass separated by 2 mm-thick spacers. Physical cross-linking of polymer solutions was then performed through a partially modified freeze-thaw (FT) process, according to Lozinsky et al. [37]. In particular, neat and oxidized PVA aqueous solutions were poured between two glass sheets separated by 2 mm-spacers, frozen at −20 °C for 24 h and then thawed at −2.5 °C for 24 h. After three FT cycles, the hydrogels were stored at −20 °C until use.

### 4.12. Mechanical Tests

Uniaxial tensile tests were carried out on a Bose ElectroForce^®^ Planar Biaxial Test Bench instrument (TA Instruments, New Castle, USA). The system was equipped with a load cell of 22 N. Rectangular samples with 1:4 width-to-free length ratio (width 5 mm × free length 20 mm) were cut from 2 mm-thick scaffolds for tensile testing. Tests were carried out up to 100% strain at a constant strain rate of 0.5%s^−1^. The samples were hydrated for the entire duration of the test by dripping physiologic solution on the sample surface. Preliminary tests pointed out an isotropic and almost-incompressible behavior, verified for each material through local strain measurements [38]. Ten test repetitions for each material were performed.

Force versus elongation data were acquired during the tests. Nominal stress σ was calculated as current force divided by the section area of the sample (initial width x thickness). Nominal strain was calculated as the ratio between the imposed elongation and the initial length of the sample. Experimental data were analyzed with a functional data analysis approach [39], by means of statistical computing software R (R version 3.6.0, The R Foundation) [40]. The raw data of each single test were preprocessed, performing a smoothing with B-splines basis. Specifically, a cubic B-spline basis with 100 internal equally spaced knots over the domain (0, 1) was used. After the preprocessing, experimental data were splint into 5 different groups according to material (PVA, OxPVA_KMnO_4__1, OxPVA_Br_2__1, OxPVA_Cl_2__1 and OxPVA_I_2__1) and functional group-specific means and standard deviations were computed.

### 4.13. Scaffold Biocompatibility

#### 4.13.1. In Vitro Cytotoxicity Assay

Neat and oxidized PVA cytotoxic effects were evaluated on the human fetal lung fibroblast cell line IMR-90 (ATCC, Manassas, VA, USA). Cells were cultured in 60 mm-petri dishes (Corning, NY, USA) using Minimum Essential Medium (MEM) (Sigma-Aldrich) supplemented with 10% fetal bovine serum (FBS) (Sigma-Aldrich), 1% L-glutamine (200 mM) (Sigma-Aldrich) and 1% penicillin (10,000 U/mL)/streptomycin (10 mg/mL) solution (Sigma-Aldrich). Cultures were maintained at 37 °C, 95% relative humidity and 5% CO_2_ changing medium every other day until the time of use. The extract test was performed on IMR-90 cells of passage 25–27 to assess the cytotoxicity of any leachable by-products from neat and oxidized PVA. To this end, PVA, OxPVA_KMnO_4__1, OxPVA_Br_2__1, OxPVA_Cl_2__1 and OxPVA_I_2__1 disks (diameter: 7 mm; thickness: 2 mm) were used to prepare soluble extracts by incubating them into IMR-90 culture medium (1 mL/100 mg disk weight), at 37 °C for 72 h. The supernatants were then collected for cell treatment.

In parallel, fibroblasts were seeded into wells of a flat-bottomed 96-well plate (Corning) at a density that would achieve confluence on attachment. After cells were let adhere for 24 h, the culture medium was aspirated and replaced with the extract media collected as previously described. The positive (cytotoxic) control was prepared by incubating cells in culture medium added with 50% DMSO, whereas the negative control was represented by untreated cultures. Cells were then incubated for a further 24 h at 37 °C in 5% (v/v) CO_2_ in air before the effect of the extracts on cell viability was determined using the MTT assay [41].

Treated and untreated IMR-90 cells were incubated with 0.5 mg/mL MTT for 4 h. Formazan precipitates were dissolved by using 2-propanol acid (0.04 M HCl in 2-propanol) and samples were analyzed with a Microplate auto reader VICTOR3™ (PerkinElmer, Waltham, MA, USA) to measure the optical density at 570 nm. The percentage of viable, metabolically active cells was calculated in comparison with the untreated control (100%) to determine the results of cytotoxicity assay.

#### 4.13.2. In Vivo Implantation

Animal procedures were carried out after obtaining the approval by the ethical committee of the University of Padua and by the Italian Department of Health (Permission code: 977/2015-PR, 21 September 2015).

Before surgery, BALB/c mice (*n* = 15) were given gas anesthesia (isoflurane/oxygen) and their dorsal cutis was shaved and sterilized with Betadine^®^ (Bayer, Leverkusen, Germany). After that, a 20 mm-lumbotomy incision was executed on the right side, by using a No. 10 surgical blade (Becton-Dickinson, Franklin Lakes, NJ, USA). Resorting to the blunt dissection technique, a subcutaneous pouch was obtained and discoidal scaffolds (diameter: 7 mm; thickness: 2 mm) of PVA, OxPVA_KMnO_4__1, OxPVA_Br_2__1, OxPVA_Cl_2__1 and OxPVA_I_2__1 (*n* = 3 for each group) were inserted in. To avoid graft displacement, samples were anchored to the latissimus dorsi muscle by using Tycron 4/0 sutures. Finally, absorbable Novosyn 4/0 sutures were used to stitch the skin. The first 5 days from surgery, the animals were administered antibiotic and anti-inflammatory therapy and were monitored during the whole recovery period.

The mice were euthanized 4 weeks from surgery by asphyxiation with carbon dioxide and implanted scaffolds were excised together with the surrounding tissues. Samples were then fixed with 10% formalin solution in neutral PBS and embedded in paraffin for histological and immunohistochemical analyses. To this end, explants were cut into 5 μm-thick serial sections, which were stained with hematoxylin and eosin (Bio Optica, Milan, Italy) according to routine protocols. Immunological characterization of the cells identified in contiguity with PVA-derived scaffolds was carried out by Dako Autostainer/Autostainer Plus (Dako, Milan, Italy), according to previously standardized protocols^11^. In particular, antigen immunolocalization on samples was performed by using anti-CD3 (A0452; Dako) and anti-F4/80 (sc-26643-R; Santa Cruz Biotechnology, CA, USA) primary antibodies, which were diluted in PBS 1:500 and 1:800, respectively. The binding between primary antibody and specific antigen was then revealed by a labeled polymer (EnVision™ FLEX-HRP; Dako) and 3,3′-diaminobenzidine (EnVision™ FLEX Substrate buffer + DAB + Chromogen; Dako). In parallel, negative controls were prepared without incubation with primary antibodies.

### 4.14. Statistical Analysis

For the characterization of oxidized PVA solutions and the cytotoxicity tests, results are expressed as means of three different experiments ± standard deviation (SD). For the statistical analysis of data, one-way analysis of variance (ANOVA) and Tukey’s multiple comparisons test were performed. Significant differences were determined by p values ≤ 0.05. Statistical analysis was performed by using Prism 8.1.0 (GraphPad Software, San Diego, CA, USA).

In parallel, to evaluate the difference in mechanical response of neat and oxidized PVA scaffolds, a functional ANOVA test was performed using the *fdatest* package in R. The latter implements the interval-wise testing procedure proposed in the literature by Pini and Vantini [42] and allows to produce a functional *p*-value which can be used to assess if the differences between the functional means of the groups is significant along their domains. The secant elastic modulus E_s_ was calculated for mean data of each material, as the slope of the straight line drawn from the origin of the stress-strain diagram and intersecting the mean curve at 10% and 20% strain. The secant elastic modulus at 10% strain for different materials was compared with ANOVA test followed by Bonferroni post-hoc test, considering a significant total *p*-value ≤ 0.05.

## 5. Conclusions

In this work, preparation of PVA-based scaffolds through partial oxidation with halogens as Br_2_, Cl_2_ and I_2_ was successfully performed for the first time in literature. In particular, Br_2_ and I_2_ seemed to better respond to the need of finding effective oxidizing agents that would allow for the minor polymer molecular weight alteration compared to KMnO_4_, with Br_2_ assuring for the most satisfactory results. All oxidized PVA hydrogels showed to preserve the high biocompatibility and atoxicity properties of the starting polymer, exhibiting mechanical behavior that suggests the possibility to realize engineered scaffolds with tunable stiffness to fulfil the demands of different injured tissues. Further studies will be focused on investigating the biological behavior of novel PVA hydrogels, to verify the acquisition of improved biodegradation ability and protein absorption/release capacity in comparison with non-modified polymer. In virtue of the insertion of carbonyl functionalities in the polymer chain through oxidation, chemically modified scaffolds will undergo specific functionalization with cell adhesion sequences and trophic factors, to evaluate their capacity to sustain cell growth and act as drug delivery systems.

## Figures and Tables

**Table 1:** Partial oxidation of PVA. Chemical schemes representing the reactions for PVA oxidation with different agents.

Oxidizing Agent	Chemical Reaction
KMnO_4_	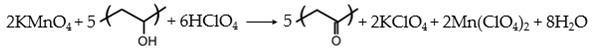
Br_2_	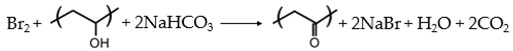
Cl_2_	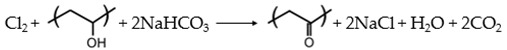
I_2_	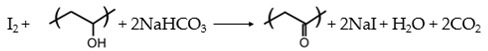

**Table 2:** Carbonyl and moisture content. The percentage of carbonyl groups (% oxidation) introduced by chemical oxidation into the polymer backbone was quantified by 2,4-dinitrophenylhydrazine (DNPH) assay. The lyophilized PVA, both in the neat and oxidized form, was evaluated for the water content (% moisture).

Polymer	% Oxidation	% Moisture
PVA	0.000	7.21
OxPVA_KMnO_4__1	0.250 ± 0.03	7.59
OxPVA_KMnO_4__2	0.560 ± 0.07	7.58
OxPVA_Br_2__1	0.244 ± 0.09	8.43
OxPVA_Br_2__2	0.576 ± 0.02	9.43
OxPVA_Cl_2__1	0.228 ± 0.04	6.52
OxPVA_Cl_2__2	0.356 ± 0.02	8.52
OxPVA_I_2__1	0.234 ± 0.05	7.86
OxPVA_I_2__2	0.490 ± 0.04	7.87

**Table 3:** Secant elastic modulus E_s_. The secant elastic modulus (with associated standard deviation SD) is calculated as the slope of the line drawn from the origin of the stress-strain diagram and intersecting the mean curve at 10% and 20% strain. Significant changes in secant elastic modulus of different hydrogels were determined by Bonferroni post-hoc test after ANOVA (*: *p* < 0.05 in comparison with neat PVA; °: *p* < 0.05 in comparison with OxPVA_KMnO_4__1; ^∆^: *p* < 0.05 in comparison with OxPVA_Cl_2__1).

	E_s_ ± SD (kPa)	
Polymer	10% Strain	20% Strain
PVA	217 ± 66	244 ± 53
OxPVA_KMnO_4__1	60 ± 30 *	70 ± 23 *
OxPVA_Br_2__1	108 ± 19 *	119 ± 16 *^,^°
OxPVA_Cl_2__1	144 ± 20 *^,^°	146 ± 19 *^,^°
OxPVA_I_2__1	90 ± 25 *^,∆^	105 ± 12 *^,∆^

## References

[1] Mandrycky C., Phong K., Zheng Y. (2017). Tissue engineering toward organ-specific regeneration and disease modeling. MRS Commun..

[2] Ahadian S., Khademhosseini A. (2018). Smart scaffolds in tissue regeneration. Regen Biomater..

[3] Kobayashi E. (2016). Challenges for Production of Human Transplantable Organ Grafts. Cell Med..

[4] Lee J.H., Kim H.W. (2018). Emerging properties of hydrogels in tissue engineering. J. Tissue Eng..

[5] Bryksin A.V., Brown A.C., Baksh M.M., Finn M.G., Barker T.H. (2014). Learning from nature-Novel synthetic biology approaches for biomaterial design. Acta Biomater..

[6] Khan F., Tanaka M. (2017). Designing Smart Biomaterials for Tissue Engineering. Int. J. Mol. Sci..

[7] Porzionato A., Stocco E., Barbon S., Grandi F., Macchi V., De Caro R. (2018). Tissue-Engineered Grafts from Human Decellularized Extracellular Matrices: A Systematic Review and Future Perspectives. Int. J. Mol. Sci..

[8] Gavasane A.J., Pawar H.A. (2014). Synthetic Biodegradable Polymers Used in Controlled Drug Delivery System: An Overview. Clin. Pharmacol. Biopharm..

[9] Baker M.I., Walsh S.P., Schwartz Z., Boyan B.D. (2012). A review of polyvinyl alcohol and its uses in cartilage and orthopedic applications. J. Biomed. Mater. Res. B Appl. Biomater..

[10] Dattola E., Parrotta E.I., Scalise S., Perozziello G., Limongi T., Candeloro P., Coluccio M.L., Maletta C., Bruno L., De Angelis M.T. (2019). Development of 3D PVA scaffolds for cardiac tissue engineering and cell screening applications. RSC Adv..

[11] Ng K.W., Torzilli P.A., Warren R.F., Maher S.A. (2014). Characterization of a microporous polyvinyl alcohol scaffold for the repair of focal articular cartilage defects. J. Tissue Eng. Regen. Med..

[12] Cutiongco M.F., Anderson D.E., Hinds M.T., Yim E.K. (2015). In vitro and ex vivo hemocompatibility of off-the-shelf modified poly(vinyl alcohol) vascular grafts. Acta Biomater..

[13] Alhosseini S.N., Moztarzadeh F., Kargozar S., Dodel M., Tahriri M. (2015). Development of Polyvinyl Alcohol Fibrous Biodegradable Scaffolds for Nerve Tissue Engineering Applications: In Vitro Study. Int. J. Polym. Mater. Polym. Biomater..

[14] Lu Y., Kong Q., Jing R., Hu X., Zhu P. (2013). Solid state oxidation of polyvinyl alcohol by hydrogen peroxide-Cu (II). Polym. Degrad. Stab..

[15] Won Y.S., Baek S.O., Tavakoli J. (2001). Wet Oxidation of Aqueous Polyvinyl Alcohol Solution. Ind. Eng. Chem. Res..

[16] Zimin Y.S., Borisov I.M., Borisova N.S., Mustafin A.G. (2013). Oxidation and Destruction of Polyvinyl Alcohol in the Aqueous Phase. Int. J. Chem. Kinet..

[17] Lin C.-C., Lee L.-T., Hsu L.-J. (2014). Degradation of polyvinyl alcohol in aqueous solutions using UV-365 nm/S2O8 22 process. Int. J. Environ. Sci. Technol..

[18] Maqsood A.M., Mohammad I., Zaheer K. (2009). Kinetics of permanganate oxidation of synthetic macromolecules poly(vinyl alcohol). Indian, J. Chem..

[19] Stocco E., Barbon S., Grandi F., Gamba P.G., Borgio L., Del Gaudio C., Dalzoppo D., Lora S., Rajendran S., Porzionato A. (2017). Partially oxidized polyvinyl alcohol as a promising material for tissue engineering. J. Tissue Eng. Regen Med..

[20] Barbon S., Stocco E., Negro A., Dalzoppo D., Borgio L., Rajendran S., Grandi F., Porzionato A., Macchi V., De Caro R. (2016). In vitro assessment of TAT-Ciliary Neurotrophic Factor therapeutic potential for peripheral nerve regeneration. Toxicol. Appl. Pharmacol..

[21] Porzionato A., Barbon S., Stocco E., Dalzoppo D., Contran M., De Rose E., Parnigotto P.P., Macchi V., Grandi C., De Caro R. (2019). Development of Oxidized Polyvinyl Alcohol-Based Nerve Conduits Coupled with the Ciliary Neurotrophic Factor. Materials (Basel).

[22] Stocco E., Barbon S., Lora L., Grandi F., Sartore L., Tiengo C., Petrelli L., Dalzoppo D., Parnigotto P.P., Macchi V. (2018). Partially oxidized polyvinyl alcohol conduit for peripheral nerve regeneration. Sci. Rep..

[23] Stocco E., Barbon S., Macchi V., Tiengo C., Petrelli L., Rambaldo A., Borean A., Capelli S., Filippi A., Romanato F. (2019). New bioresorbable wraps based on oxidized polyvinyl alcohol and leukocyte-fibrin-platelet membrane to support peripheral nerve neurorrhaphy: Preclinical comparison versus NeuraWrap. Sci. Rep..

[24] Baruah S.D., Laskar N.C. (1996). Relationship between Molecular Weight and Viscosity for Polydispersed Poly(n-docosyl acrylate). Polym. J..

[25] Stocco E., Barbon S., Dalzoppo D., Lora S., Sartore L., Folin M., Parnigotto P.P., Grandi C. (2014). Tailored PVA/ECM scaffolds for cartilage regeneration. Biomed. Res. Int..

[26] Stocco E., Barbon S., Radossi P., Rajendran S., Dalzoppo D., Bortolami M., Bagno A., Grandi F., Gamba P.G., Parnigotto P.P. (2016). Autologous chondrocytes as a novel source for neo-chondrogenesis in haemophiliacs. Cell Tissue Res..

[27] Grandi F., Stocco E., Barbon S., Rambaldo A., Contran M., Fascetti Leon F., Gamba P., Parnigotto P.P., Macchi V., De Caro R. (2018). Composite Scaffolds Based on Intestinal Extracellular Matrices and Oxidized Polyvinyl Alcohol: A Preliminary Study for a New Regenerative Approach in Short Bowel Syndrome. Biomed. Res. Int..

[28] Dash S., Patel S., Mishra B.K. (2009). Oxidation by permanganate: Synthetic and mechanistic aspects. Tetrahedron.

[29] Ye B., Li Y., Chen Z., Wu Q., Wang W., Wang T., Hu H. (2017). Degradation of polyvinyl alcohol (PVA) by UV/chlorine oxidation: Radical roles, influencing factors, and degradation pathway. Water Res..

[30] Moorthy J.N., Singhal N., Senapati K. (2007). Oxidative cleavage of vicinal diols: IBX can do what Dess–Martin periodinane (DMP) can. Org. Biomol. Chem..

[31] Kazimierska-Drobny K., El Fray M., Kaczmarek M. (2015). Determination of mechanical and hydraulic properties of PVA hydrogels. Mater. Sci. Eng. C Mater. Biol. Appl..

[32] Zhang Y.R., Xu K.J., Bai Y.L., Tang L.Q., Jiang Z.Y., Liu Y.P., Liu Z.J., Zhou L.C., Zhou X.F. (2018). Features of the volume change and a new constitutive equation of hydrogels under uniaxial compression. J. Mech. Behav. Biomed. Mater..

[33] Liu K., Ovaert T.C. (2011). Poro-viscoelastic constitutive modeling of unconfined creep of hydrogels using finite element analysis with integrated optimization method. J. Mech. Behav. Biomed. Mater..

[34] Karimi A., Navidbakhsh M., Beigzadeh B. (2014). A visco-hyperelastic constitutive approach for modeling polyvinyl alcohol sponge. Tissue Cell.

[35] Bernard M., Jubeli E., Pungente M.D., Yagoubi N. (2018). Biocompatibility of polymer-based biomaterials and medical devices-regulations, in vitro screening and risk-management. Biomater. Sci..

[36] Sajonz P., Bookalam J., Miller R.A. (2006). Separation of Periodate, Iodate and Iodide on a C-18 Stationary Phase. Dependence of the Retention on the Temperature and Solvent Composition. Monitoring of an Oxidative Cleavage Reaction. Chromatographia.

[37] Lozinsky V.I., Solodova E.V., Zubov A.I., Simenel I.A. (1995). Study of Cryostructuration of Polymers Systems. XI. The Formation of PVA Cryogels by Freezing-Thawing the Polymer Aqueous Solutions Containing Additives of Some Polyols. J. Appl. Polym. Sci..

[38] Todros S., Pianigiani S., de Cesare N., Pavan P.G., Natali A.N. (2019). Marker tracking for local strain measurement in mechanical testing of biomedical materials. J. Med. Biol. Eng..

[39] Ramsay J.O., Silverman B.W. (2005). Functional Data Analysis.

[40] R Core Team (2018). R: A Language and Environment for Statistical Computing.

[41] Hansen M.B., Nielsen S.E., Berg K. (1989). Re-examination and further development of a precise and rapid dye method for measuring cell growth/cell kill. J. Immunol. Methods.

[42] Pini A., Vantini S. (2017). Interval-wise testing for functional data. J. Nonparamet. Stat..

